# Psychological Emotion and Behavior Analysis in Music Teaching Based on the Attention, Relevance, Confidence, and Satisfaction Motivation Model

**DOI:** 10.3389/fpsyg.2022.917476

**Published:** 2022-06-28

**Authors:** Dong Li

**Affiliations:** Conservatory of Music, Nanjing Normal University, Nanjing, China

**Keywords:** ARCS motivation model, music teaching, psychological emotion, behavioral motivation, inquiry teaching, motivational strategy

## Abstract

The abbreviation ARCS in the ARCS motivational model comprises the first letters of four English words: Attention, Relevance, Confidence, and Satisfaction. The ARCS motivation model is based on a systematic and easy-to-operate motivation theory. Many research studies have verified the applicability and effectiveness of the ARCS model in education worldwide. The proposed optimized ARCS motivation model takes the traditional ARCS motivation model and systematically optimizes it to make it suitable for the coding of data from videos of music classes. The case analysis method selected 14 high-quality music lessons as the research object. The optimized ARCS model involves text conversion, coding, and statistical analysis of the transcripts of videos of high-quality music classes. The data analysis was used to analyze the characteristics of students’ emotional and behavioral motivation incentive strategies in each section of the ARCS motivation model in combination with specific high-quality case lesson fragments. This paper summarizes ways and means of using a motivational approach in the teaching of quality music classes, providing theoretical support for the practice of music teaching.

## Introduction

Motivation is the internal power that stimulates and maintains individual psychological activities guided by goals and objects. Motivation exists in most human behaviors and in learning. Keller, an American psychology professor, believes that non-intelligence factors determine the learning effect, and learning motivation is the core non-intelligence factor (Keller, 1987). Therefore, learning motivation has a substantial impact on learning results. The unitary music teaching mode is one of the reasons for the continuous decline in music students’ learning motivation. Applying the attention, relevance, confidence, and satisfaction (ARCS) motivation design model in music teaching provides teachers with diversified teaching forms. When music teachers are planning classes, to optimize the quality of their teaching, they should fully understand their teaching materials and their students’ emotional and behavioral motivation (Zhou and Tang, 2020).

In 1984, Keller first introduced the origin, significance, and theoretical basis of the ARCS motivation model and introduced the application of the ARCS motivation model to teaching design (Liao et al., 2020). In 1988, Keller and Suzuki started research on the practical application of the ARCS motivation model (Christine et al., 2015). In 1996, Keller reported relevant strategies of motivation design based on multimedia features. Since then, the ARCS motivation model has been applied in many countries and fields worldwide (Dörnyei and Al-Hoorie, 2017). Since 2013, research on the ARCS motivation model in China has gradually gained attention, and the practice of various teaching forms has been widely used (He, 2021). In the last 10 years, research on the ARCS motivation model has mainly focused on optimizing the model itself and the universality of its application. Compared with other theories, this model is more practical and suitable for education (Li, 2018; Li et al., 2018).

In this study, the traditional teaching contents of the ARCS motivation model and analyzes the problems of the students’ learning motivation for the music teaching of middle school students emotion and behavior, to the traditional ARCS motivation model coding, system optimization, set up the corresponding teaching strategies, and apply it to the practice of music teaching, arouse the students’ interest in learning music and motivation. It provides theoretical support for music teaching practice.

## Optimization of the Attention, Relevance, Confidence, and Satisfaction Coding System

The ARCS coding system is composed of the first letters of four English words: Attention, Relevance, Confidence, and Satisfaction (Angelo, 2017). The ARCS motivation model includes four first-level strategies: A: attention strategy, R: immediate strategy, C: self-confidence strategy, and S: satisfaction strategy. Through the theoretical research and development status analysis of the ARCS motivation model, the ARCS motivation model is built based on comprehensive learning motivation theory, endowed with macro and broad applicability. The ARCS motivation model can be applied to more fields, but it does not apply to fixed subjects. It is necessary to systematically optimize the coding of traditional ARCS according to the characteristics of music teaching and the emotion and behavior of students in music learning.

### Attention Strategy

Attention is the primary factor in stimulating and maintaining learning motivation and the prerequisite for effective learning. Although cultivating and preserving the learners’ learning motivation is a form of systematic engineering that involves a wide range and runs through the learning process, attracting and supporting learners’ attention is the first and most critical step in stimulating and maintaining learners’ learning motivation.

Attention in music teaching has the characteristics of directivity and concentration, including intentional attention and unintentional attention. Changing how media are presented generally only attracts involuntary attention, while exposing learners to interesting problems will attract deliberate attention. Teaching staff should use students’ conscious awareness to stimulate and maintain learning motivation in the teaching process. The primary purpose of instructional designers is to arouse learners’ curiosity and make them choose to direct their attention to learning activities consciously or unconsciously. Table 1 shows the optimized coding system for the attention strategy.

**TABLE 1:** Attention strategy coding system after optimization.

First strategy	Secondary strategy	Motivational strategies
A Attention	A1 conflict	A1-1: Provide facts that conflict with established experience
		A1-2: Provide examples that do not seem to exemplify a concept
		A1-3: Offer two equally reasonable examples, only one of which is true
	A2 concrete	A2-1: Use concrete examples or intuitive ways to explain abstract knowledge of music
		A2-3: Use anecdotes, case studies, biographies, etc., related to music teaching content
	A3 change	A3-1: Vary your style, use body language, etc.
		A3-2: Change how teaching is organized
		A3-3: In PowerPoint, keep the picture plain, the content simple, and the emphasis on highlighting. Use different fonts to emphasize the title, extracts, rules, etc.
	A4 humor	A4-1: Use humorous language or appropriate humorous metaphors
	A5 question	A5-1: Set up problem situations
		A5-2: Include a short questioning session
	A6 participation	A6-1: Involve students interactions

### Relevance Strategy

Immediacy refers to the relationship between learning content and learning objectives. When the students’ attention is caught, they may ask, “How does this relate to our goals?” According to the expectation and value theory, the degree of correlation between learning objectives and learning content can determine the depth of learning content (Chen, 2019). Therefore, when learners think that the learning content is closely related to the learning goal, they will usually show more interest, thereby encouraging the positive development of learning motivation.

There are two kinds of immediacy in music teaching: objective-oriented proximity and process-oriented closeness. Purpose-oriented realism is instrumental and practical. Students’ learning motivation is stimulated if the learning content helps the learners achieve an important goal. Process orientation considers the learners’ feelings in the learning process and emphasizes that learners should feel that the learning itself is consistent with their personal characteristics. An excellent emotional experience will stimulate learning motivation when learners think they have a role in the learning process (Yi and Wu, 2020). Table 2 shows an optimized coding system for relevance.

**TABLE 2:** Relevance strategy coding system after optimization.

First-level strategy	Secondary strategy	Motivational strategies
R Relevance strategy	R1 experience	R1-1: Show the relationship between the content of the teaching
		R1-2: By analogy with experience, relate the knowledge to real life
	R2 learning value	R2-1: Show the relationship between current value, learning content, and subsequent courses
		R2-2: Show the future value, the connection between learning content and students’ future lives
	R3 requirement matching	R3-1: Encourage students to compete against others or against standards
		R3-2: Meet students’ affinity needs. Build trust between teachers and students
	R4 model	R4-1: Introduce the anecdotes about the people behind the music

The essential difference between the two kinds of immediacy is that purposeful immediacy locates the motivation source on the setting of external utilitarian goals. Although appropriate goal setting is conducive to the internalization of learning motivation, the goal is still a part of external incentives, and its motivational effect will not last long. However, process immediacy emphasizes the pleasure of the learning process and makes the initially complex learning become fun rather than a burden, thereby combining the two kinds of proximity to achieve the best effect.

### Confidence Strategy

Confidence is the third element that inspires and sustains learning motivation. Confidence means that students believe they have a distinct possibility of success and can complete learning tasks and achieve learning goals. When learners become interested in learning and developing their skills, Keller thinks they need to be motivated to help them overcome difficulties and obstacles. They need to establish the confidence of “believing that they can complete tasks.” Otherwise, they may give up on the learning tasks. According to Room’s expectation theory, people are always eager to meet particular needs and achieve specific goals. This goal has an impact on stimulating people’s motivation. The size of the motivational power depends on the target value (Valence) and expectation rate (Expectancy). The formula is: MF = E – V. MF is the Motive Force or strength of motivation. E is Expectancy, meaning the probability that a specific action may lead to realizing the desired goal. V is Valence, referring to people’s emphasis on a particular destination or achievement (Peng, 2020). Table 3 shows the optimized coding system for the confidence strategy. The expected rate is proportional to the intimate connection with self-confidence. If learners have no confidence, it is E = 0, so even if learning is itself titer enabled him to get enough interests, V-, continue to promote learning motivation MF is still zero. The vital role of confidence in learning motivation cannot be ignored.

**TABLE 3:** Confidence strategy coding system after optimization.

First strategy	Secondary strategy	Motivational strategies
C Confidence strategy	C1 learning Requirements	C1-1: Clearly express the requirements for learning success, clearly state students’ learning objectives
	C2 expectations	C2-1: Encourage students to express their trust and expectations
	C3 attribution	C3-1: Guide students to correct attributions when appropriate

### Satisfaction Strategy

Satisfaction is the psychological experience that learners get when their learning results are consistent with their positive expectations. Satisfaction is the fourth element of the ARCS motivation design model and an essential condition for learners to generate continuous learning motivation (Zhao and Zhang, 2018). In learning, students should have learning results and should experience a sense of accomplishment after completing the task. The factors that affect satisfaction are reinforcement and feedback, internal reward, and cognitive evaluation. Cognitive evaluation refers to the internal process of evaluating the consequences of behavior according to one’s expectations. If a person has a high expectation of success, they may not feel satisfied with the result after completing the task. Conversely, if the level of expectation is low, the student may experience satisfaction.

The ARCS motivation design model can be regarded as a process. The first element in stimulating a person’s learning motivation is to attract attention and interest in learning, to make the learner understand that the accomplishment of the task depends on them. Then, the learner needs to feel confident in his ability to perform the task. Finally, the learner should experience a sense of achievement and satisfaction (Xu, 2020). Table 4 shows the optimized coding system for satisfaction. Therefore, attention, relevance, confidence, and satisfaction are a whole. There is no primary or secondary aspect, and the lack of any one element may cause learners to lose learning motivation.

**TABLE 4:** Satisfaction strategy coding system after optimization.

First strategy	Secondary strategy	Motivational strategies
S Satisfaction strategy	S1 internal reinforcement	S1-1: Use knowledge to solve practical problems in life
		S1-2: Praise the students verbally and agree with their opinions
		S1-3: Help students who have not completed a task
	S2 external reinforcement	S2-1: Congratulate students for good behavior
		S2-2: Give personal attention to students

The ARCS motivation model provides music teachers with the basic teaching design ideas. First, they need to attract students’ attention. Second, teaching content should be closely linked to students’ interests and needs. Next, students should be helped to build self-confidence and to be convinced that they can succeed. Finally, teachers should strengthen students’ satisfaction, thereby maintaining their learning motivation at a high level.

## Case Analysis

In the process of music teaching as a whole, teachers use various teaching strategies and means to stimulate and maintain students’ learning motivation. Different teachers use different teaching strategies. In this study, teachers used the improved ARCS motivation model as a coding system to study the coding analysis of quality music lessons. The statistical analysis of music teachers’ high-quality classroom teaching strategies could provide valuable references and suggestions to inspire chemistry teachers to stimulate and maintain students’ learning motivation.

### Research Object and Material Selection

#### Research Object

Classroom teaching is a bilateral interactive activity between teachers and students in class. Classroom teaching is the most effective way for students to acquire knowledge. It enables teachers to pass on culture and to promote the all-round development of their students. In classroom teaching, teachers should enable students to master knowledge skills, and they should cultivate students’ appropriate emotional attitudes and values. The process of classroom teaching is a unique cognitive activity involving guidance, simplicity, and restriction. Teacher-student communication is the background to the promotion of the means and activities necessary for students to pursue and realize the target values for their overall development and for their physical and mental development. Teachers stimulate and maintain students’ learning motivation in classroom teaching activities, and they select students’ psychological emotions and behavior rules in participating in class as research objects.

#### Research Material Selection

Based on the requirements of high-quality education in recent years, the music lessons selected by the research institute must have a particular demonstration role and research value. The conclusions could enlighten the majority of teachers. A quality class is a class carefully designed and arranged by an excellent team of teachers. It condenses the wisdom and experience of senior teachers, embodies the most advanced teaching concepts, and the judges are highly recognized. High-quality classes have excellent guidance and reference significance for all subject teachers.

Fourteen quality music lessons were selected to explore the teaching strategies used to stimulate students’ learning motivation and their emotional and behavioral feedback in music class. The 14 selected classes cover spectrum recognition, tone pronunciation, musical instruments, etc. Insofar as possible, the research scope covers all the content of the music classes and accurately analyzes the teachers’ strategies in stimulating students’ learning motivation and their emotions and behavior in class. Table 5 shows the selected categories.

**TABLE 5:** Fourteen high school quality music lesson examples.

Class number	Lesson topic	Category
Case 1	I’m a Little Musician	Singing class
Case 2	I am a Grassland Herdsman	Singing class
Case 3	The Magpie Drilled through the Fence	Appreciation class
Case 4	Be Like a Green Pine on the top of Mount Tai	Appreciation class
Case 5	Bing Bong Variations	Singing class
Case 6	Typewriter	Appreciation class
Case 7	Happy Little Flute	Singing class
Case 8	Knitting Basket	Comprehensive course
Case 9	Withered Stalk a Communal	Comprehensive course
Case 10	Beijing Tone	Appreciation class
Case 11	The Song of the Forest	Appreciation class
Case 12	Sorcerer’s Disciple	Appreciation class
Case 13	The Song of the Forest	Singing class
Case 14	Riverstones	Singing class

### Material Processing

#### Transcription of Music Teaching Videos

This paper transcribed the videos of the 14 selected high-quality teaching classes word for word to make a comprehensive and detailed analysis of the students’ emotions and behavior based on the motivation model in the class. SPEED CODINGLF software was used to complete the transcripts of the classroom videos (Qi et al., 2020). The software directly converts the classroom videos into text so that the audio content is identified clearly and accurately and presented in text form. After the transcripts were completed, teachers conducted observation and analysis using a 60-s time unit and modified any inaccurate transcription. The teachers identified students’ feedback in class and noted students’ emotions (language, facial expression, voice, and intonation) and behaviors (movement, demonstration, interactions).

For the integrity of analysis, if a sentence exceeded the specified range of 60 s, it was directly counted as being 60 s and was not extended to the next unit.

#### High-Quality Course Coding Process

In the transcripts of the 14 lessons, the language of some students was not clear and accurate, and there was a high level of colloquial language. Word processing software ensured clear and concise text presentation and smooth sentences, and the teachers modified unclear or idiomatic expressions to preserve the original expressions, behaviors, and emotions. The classroom videos were coded according to the motivational strategies of the optimized ARCS motivational model.

In classroom teaching, a student’s learning behavior involves not just one motivational stimulation strategy but it is likely to involve multiple motivational stimulation strategies. Therefore, there are several different results in the coding according to the optimized ARCS motivation model within the same period. The teachers analyzed the classroom videos, identifying the motivational strategies and coding them according to the ARCS motivation model involved in the class.

Take “Happy Little Flute” (Case 7) as an example. At the beginning of class, in a loud voice, the teacher asks the students, “Do you want to live a happy life?” Not the opening words of teachers, and chose the “good life” everyone want to stimulate students’ interest in learning, to the other coding for “room A41 teacher use humor language” this problem, students answer “to” actively. Then temper, teachers put forward “how to make you feel happy,” teachers to create scenes. The selected settings related to students’ lives; they were in line with current social hot spots and could make students more eager to solve the problems closely related to motivation. The sentence is coded as “R2-1 future value, R3-1 teachers motivate students to solve problems.” The students discussed the content of the “Happy Little Flute” lesson and were motivated to experience good emotions and achieve the learning objective. The sentence is coded as “C1-1 learning requirements: clear learning objectives.” During the class, the teacher gave timely verbal praise and affirmation to the students to reward their cooperative behaviors and emotions, and the students were given the appropriate encouragement when they sang along. The coding results for this time node are as follows: “C2-1 encourages students and expresses their support for and trust in the students,” “C3-1 guides students to correct attributions when appropriate,” and “S1-2 gives students oral praise and agrees with students’ opinions when appropriate.”

### Data Processing and Analysis

#### Reliability and Validity Analysis of Attention, Relevance, Confidence, and Satisfaction Coding System

The coding used for the music classroom video studied in this paper is manual coding, which has inevitable artificial subjectivity. A reliability and validity test analysis of the coding was necessary to ensure the accuracy and reliability of the final coding results (Thomas, 2017). The number of codes in this paper is too large to test all the coding results, so one teaching clip was randomly selected from each classroom teaching video, 14 learning clips in total. The data would be too tedious if we were to code all the motivational strategies involved in each teaching segment. Therefore, we only observe one of the most important motivational strategies in each teaching segment. “An attention strategy” was observed in teaching segments 1–4; “R relevance strategy” was observed in teaching segments 5–8: “C confidence strategy” was observed in teaching segments 9–11; and “S satisfaction strategy” was observed in teaching segments 11–14. If no first-level strategy was observed in the selected segment, the next time node was selected, and the sequential deduction was not chosen randomly again. The 14 extracted fragments were recoded and tested according to the optimized ARCS system and provisions, and the coding results were analyzed and compared. Table 6 shows the coding results for the 14 lessons. A is the author, and B and C are two graduate students of different grades and genders. Having three people coding together ensures that data will not result in incorrect conclusions arising from gender, age, and other subjective factors.

**TABLE 6:** Test data of attention, relevance, confidence, and satisfaction (ARCS) motivation model after optimization.

Cases of class number	Cases of lesson topic	A coding results	B coding results	C coding results
A Attention	I’m a Little Musician	A2-1	A3-1	A2-1
	I am a Grassland Herdsman	A3-2	A3-2	A3-2 A3-3
	The Magpie Drilled Through the Fence	A3-3	A3-3	A3-3
	Be like a Green Pine on the Top of Mount Tai	A2-1 A3-2 A5-2	A2-1 A6-1 A3-2	A2-1 A3-2
R Relevance strategy	Bing Bong Variations	R1-1 R6-1 R4-2	R1-1 R4-1	R4-1 R6-1
	Typewriter	R1-2	R1-2	R1-2
	Happy Little Flute	R4-1	R4-1	R4-1
	Knitting Basket	R1-1 R1-2	R1-1	R1-1
C Confidence strategy	Withered Stalk a Communal	C1-1	C1-1 C2-1	C1-1
	Beijing Tone	C1-1	C1-1	C1-1
	The Song of the Forest	C2-1 C3-1	C2-1	C2-1 C3-1
S Satisfaction strategy	Sorcerer’s Disciple	S1-1 S1-2	S1-2 S1-2	S1-1
	The Song of the Forest	S1-2	S1-2 S2-2	S1-2 S2-1
	Riverstones	S1-2	S1-1 S1-2	S1-1 S1-2

The coding results of the two graduate students were compared with those of the author. They qualified no controversial encoding results since the first-level policy encoding before. After the discussion and consensus of different coding results, two significant differences remained, making the consistency an acceptable 95.23%. The optimized ARCS motivation model can be used as the basis for the analysis of research on high-quality classes (Guo, 2019).

#### Analysis of Attention, Relevance, Confidence, and Satisfaction Statistical Results

After coding the 14 extracted lessons and producing detailed statistics for each speech unit, the coding results were sorted into the second strategy, resulting in a quantitative summary of the students’ emotional and behavioral performance during high-quality music lessons. Table 7 shows the specific results.

**TABLE 7:** Statistics for attention, relevance, confidence, and satisfaction (ARCS) motivational strategies.

First strategy	Secondary strategy	Number of uses	Total	Percentage
A Attention	A1 conflict	68	1,723	59.08%
	A2 concrete	571		
	A3 change	402		
	A4 humor	54		
	A5 question	593		
	A6 participation	35		
R Relevance strategy	R1 experience	189	526	18.03%
	R2 learning value	139		
	R3 requirements matching	173		
	R4 model	25		
C Confidence strategy	C1 learning requirements	164	272	9.33%
	C2 expectations	30		
	C3 attribution	78		
S Satisfaction strategy	S1 internal reinforcement	214	398	13.65%
	S2 external reinforcement	174		

Table 7 shows that “A attention strategy” is the most frequently used strategy by teachers in class, accounting for 59.08% of the total, indicating that in music classroom teaching, teachers use many strategies to attract and maintain students’ attention. Focusing students’ attention on the classroom is the most frequently used strategy to stimulate and sustain students’ learning emotion and behavior motivation, followed by the “R relevance strategy” (about 18.03%) and the “S satisfaction strategy” (about 13.65%). “R relevance strategy” is related to students’ previous knowledge and experience. Music is a subject that is very close to life, and teachers use many examples related to students’ learning life in class to stimulate students’ interest in learning. “S satisfaction strategy” mainly focuses on the interaction between students and their emotional and behavioral interactions, which teachers praise in a timely and appropriate manner. The “C confidence strategy” was used relatively few times, accounting for a small proportion of the whole, about 9.33%.

### Lesson Case Analysis Using Attention, Relevance, Confidence, and Satisfaction

#### Attention

**FIGURE 1:**
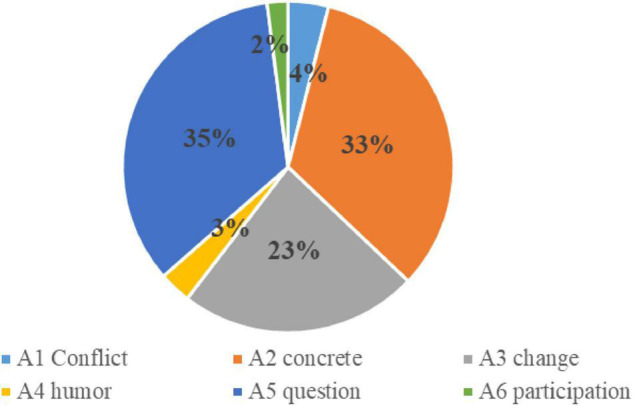
The percentage of secondary policy usage in attention strategy.

How do high-quality teachers use attention strategies in classroom teaching to stimulate students’ learning motivation? The following is a detailed analysis of specific teaching fragments from high-quality classes. Note that the total number of strategies used is 1,723. Figure 1 shows the percentage of secondary systems.

A1 involved the use of conflict policy 68 times. They accounted for 4% of attention policies. When students are presented with conflicts with their life experiences, providing two equally reasonable but only one correct example will attract students’ attention and make them think more positively and judge right from wrong using what they have learned. During the recording of the 14 class videos, teachers of high-quality classes used strategies such as inducing cognitive conflict to stimulate students’ interest in learning.

A2 involved the use of a materialization strategy 571 times, accounting for 33% of attention strategies. On the whole, motivation incentive strategies were used more often. Cognitive theory holds that, in classroom teaching, teachers should allow students to learn in a realistic environment. Simply teaching could easily cause students to find it difficult to integrate their learning emotions. Students are less motivated to learn. Visual aids and examples attract students’ attention, concretize the concepts in lyrics, allow them to visualize notes, and connect the music world with students’ lives.

A3 change involved the frequent use of motivation incentive strategy. It can be easy for students to pay attention consciously in class, but they find it difficult to pay attention for long periods. According to the ARCS motivation model, to maintain students’ attention, teachers need to constantly change their style of expression. They need to use body language and to change how they organize their teaching so that they can trigger students’ concentration.

A4 used the use of humor as a strategy 54 times, accounting for 3% of attention strategies. The overall frequency of use was moderate. Humorous people often have a wide range of knowledge and creative expressions. Amusing behavior and expressions allow students to easily accept knowledge and to be relaxed and receptive. Humorous teachers attract the attention of students, stimulate their interest in learning, and make it easy to acquire knowledge.

A5 involved the frequent use of query strategy, 593 times, accounting for about 35% of the attention strategies. Quality classes usually begin by creating problem situations for students. Active thinking is the process through which people constantly discover and solve problems. The core of the questioning strategy is the new problem presented. It should be in conflict with the students’ actual knowledge and experience, resulting in students’ mental imbalance and thinking motivation.

In A6, student participation in classroom teaching activities accounted for 2% of attention strategies. The greater the degree of involvement, the more active the students, and the stronger the students’ sense of a dominant status experience. Enhanced student subjective consciousness further improves enthusiasm and participation autonomy. This item accounts for the lowest proportion and focuses on music curriculum optimization and improvement.

#### Relevance Strategy

**FIGURE 2:**
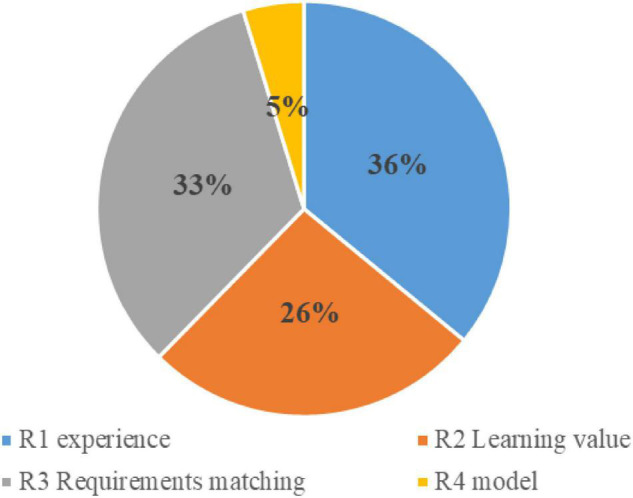
Percentage of secondary strategy usage in relevance strategy.

When students fail to realize the connection between music and life, they ask, “Why should we study music?” The problem is “immediacy.” R is used 526 times, accounting for 18.13% of the total times. Figure 2 shows the proportion of secondary strategies.

R1 experience accounted for 36% of the total use times. Constructivist theory holds that students do not enter the classroom knowing nothing. Students have already acquired rich knowledge and experience in daily life, very important for their learning of content. With a knowledge foundation, students can quickly enter the state of learning. Comparing the teaching process, the teaching content, and the actual life experience can attract students’ attention and enable them to understand new concepts.

R2 learning value accounted for 26% of the total use times. When students think that the knowledge does not apply in life, they are not able to appreciate the importance of music. Music comes from life and is the crystallization of life. Instead of having students memorize lyrics and tones. The value of music is the motivation to stimulate students’ interest in learning.

R3 requirements match 33% of the time. Teachers can attend to the needs of students and try their best to meet those needs. When the needs of students are met, the students will inevitably have greater interest in the classroom, and it will be easier to stimulate their learning motivation. “Please answer,” “Very good,” “Please sit down,” and other such phrases will invariably close the distance between teachers and students, meet the needs of the students, and establish mutual trust between teachers and students. When students feel that the instructor is easy to get to know, they will be more motivated to learn.

R4 model accounted for 5% of the total use times. Students cannot appreciate the personal charm of artists without understanding their spirit and courage in pursuing their art. Teachers’ timely introduction of events and people will arouse students’ curiosity and interest in exploring the art world and learning more actively.

#### Confidence Strategy

**FIGURE 3:**
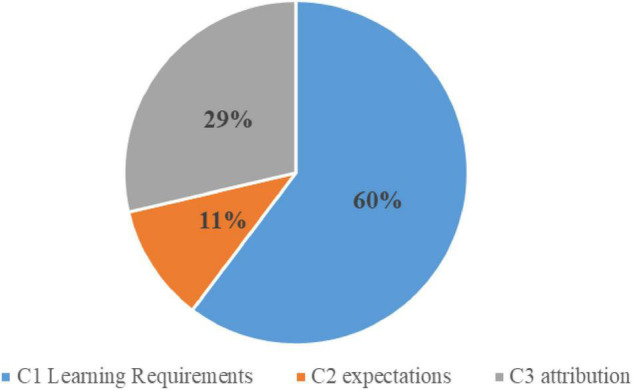
Percentage of secondary strategy usage in confidence strategy.

Confidence strategy was used 272 times, accounting for 9.33% of the total use times. Figure 3 shows the percentage of secondary strategies.

According to the ARCS motivation model, the following aspects can enhance students’ self-confidence.

C1 precise learning requirements account for 60% of the total use times. Classroom teaching objectives refer to the expected results of teaching activities: the concretization of educational purposes, teaching goals, curriculum objectives, and the requirements and standards for teachers to complete teaching tasks. It is not difficult for teachers to set classroom teaching objectives, but it is difficult for students to understand the requirements of teachers and to act on them.

C2 expectation accounted for 11% of the total use times. Every student is afraid of failure and wants to do their best. While stimulating and maintaining students’ learning motivation, teachers should think about how to develop students’ self-confidence. Appropriate encouragement could help students overcome difficulties with confidence. Proper expectations mean that the teachers’ goals are of a certain level and the students are capable of completing the tasks set. Teachers should encourage students appropriately and believe that the students can succeed.

C3 attribution accounted for 29% of the total use times. Correct attribution helps clarify the causal relationship between teachers and students’ behaviors, examine the differences between students’ behaviors and their psychological and behavioral results, predict learning motivation, improve learning behavior, and improve learning effectiveness. Correct attribution is also helpful in enhancing students’ self-awareness. Teachers should guide, attend to, and train students’ proper attribution for negative reasons.

#### Satisfaction Strategy

**FIGURE 4:**
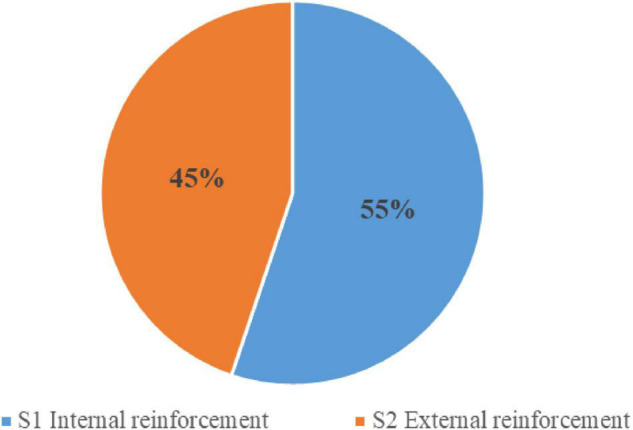
Percentage of secondary strategy usage in satisfaction strategy.

S satisfaction strategy was used 398 times in total, accounting for about 13.65% of the total use times. Figure 4 shows the percentage of secondary strategies.

When students make some effort to get results consistent with expectations, they will be satisfied. This satisfaction will further stimulate students to produce continuous, independent, in-depth learning motivation, resulting in new learning behavior.

S1 reinforcement of internal motivation accounted for 55% of the total use times. Internal motivation refers to people’s spontaneous cognition of engaging activities. There is no need to stimulate internal motivation. It is generated by the students themselves, and teachers need to think about maintaining and enhancing students’ inner motivation. In class, students can use the knowledge learned in the class to solve problems in actual production and life without the help of others, and this will strengthen students’ internal motivation. Their interest in learning will naturally become higher and higher.

S2 enhanced external motivation accounted for 45% of the total use times. External motivation is the motivation induced by stimuli other than the individual’s activities. In the classroom, external reinforcement is nothing more than the correct feedback behavior of teachers at the school. Correct feedback from teachers can make it clear to students whether their way of thinking and behaving are accurate. They can fully mobilize students’ enthusiasm, improve their interest in learning, and enhance their learning motivation.

## Conclusion

By grasping the basic theory and the characteristics of the music discipline, Keller’s ARCS motivation was coded and optimized to establish a motivation coding system suitable for music classes. The optimized ARCS motivation model was used to transcribe and encode 14 high-quality music class videos, and the optimized ARCS motivation model was analyzed and verified. The quantitative analysis, combined with the qualitative research, allowed the following conclusions to be drawn.

(1) Many motivational strategies teachers use in the 14 high-quality music lessons conform to the motivational approach in the ARCS motivational model. The statistics show that teachers mostly use an attention strategy in class, indicating that teachers use many strategies to attract and maintain students’ attention and concentration in class is the effort to stimulate and sustain students’ learning motivation.

(2) R intimacy strategy (about 18.03%) and S satisfaction strategy (approximately 13.65%) were second only to A in frequency. “R vital strategy” is related to a student’s previous knowledge and life experience. Music is a subject close to life, and teachers often use examples related to students’ learning and life in class to stimulate students’ interest in education. “S satisfaction strategy” mainly focuses on teachers’ timely praise after students’ interactions.

(3) C confidence strategy accounted for a small proportion of the whole, about 9.33%, and was rarely used. It is the crucial index for further optimization of the model.

(4) The optimized ARCS motivation model is suitable for music teaching. It can help teachers motivate students more effectively in classroom teaching. The studies and practice cases prove the teaching strategies of the model. It is a practical basis for stimulating students’ emotions and behavior in modern music teaching classrooms.

## Data Availability Statement

The raw data supporting the conclusions of this article will be made available by the authors, without undue reservation.

## Ethics Statement

The studies involving human participants were reviewed and approved by the Ethics Committee of Nanjing Normal University. Written informed consent for participation was not required for this study in accordance with the national legislation and the institutional requirements.

## Author Contributions

The author confirms being the sole contributor of this work and has approved it for publication.

## Conflict of Interest

The author declares that the research was conducted in the absence of any commercial or financial relationships that could be construed as a potential conflict of interest.

## Publisher’s Note

All claims expressed in this article are solely those of the authors and do not necessarily represent those of their affiliated organizations, or those of the publisher, the editors and the reviewers. Any product that may be evaluated in this article, or claim that may be made by its manufacturer, is not guaranteed or endorsed by the publisher.
